# Biomechanical and Physiological Variables in Dynamic and Functional Balance Control during Single-Leg Loading in Individuals with Chronic Ankle Instability: A Scoping Review

**DOI:** 10.3390/sports12080224

**Published:** 2024-08-16

**Authors:** Chairat Phuaklikhit, Thanwarat Junsri, Seiji Saito, Satoshi Muraki, Ping Yeap Loh

**Affiliations:** 1 Graduate School of Design, Kyushu University, Fukuoka 815-8540, Japan; chairat.p@rsu.ac.th; 2 Faculty of Physical Therapy and Sports Medicine, Rangsit University, Pathum Thani 12000, Thailand; 3 Faculty of Computer Science and Systems Engineering, Okayama Prefectural University, Soja 719-1197, Japan; 4 Faculty of Design, Kyushu University, Fukuoka 815-8540, Japan

**Keywords:** CAI, dynamic balance, electromyography, force plate, functional balance, inertial measurement unit, single-leg stance, ultrasonography

## Abstract

Background: This scoping review summarizes the tasks and outcomes in dynamic and functional balance assessments of individuals with chronic ankle instability, focusing on the physiological and biomechanical characteristics. Method: A comprehensive literature search was conducted in PubMed, Scopus, Web of Science, and MEDLINE databases in September 2023 and revised in April 2024. Studies evaluating dynamic and functional balance in chronic ankle instability using clinical tests, as well as biomechanical and physiological outcomes, were included. Results: Out of 536 publications, 31 met the screening criteria. A history of ankle sprain was the main focus of the inclusion criteria (28 articles, 90%). The star excursion balance test, emphasizing maximum reach distance, was the most common quantitative task (12 articles, 66%). Physiological data mainly came from electromyography studies (7 articles, 23%), while biomechanical variables were often assessed through center of pressure studies using force plates (17 articles, 55%). Conclusions: The preferred quantitative clinical assessment was the star excursion balance test, focusing on normalized reach outcomes. Qualitative functional balance assessments emphasize landing activities and center of pressure displacement. Electromyography is commonly used to analyze the tibialis anterior and peroneus longus muscles. However, there is a lack of qualitative data on dynamic balance control, including morphological characteristics and the center of mass adaptation.

## 1. Introduction

Lateral ankle sprain is a prevalent injury across all populations, especially among highly active individuals. Approximately 70% of patients develop chronic ankle instability (CAI) [1], leading to feelings of instability or “giving way” and recurrent sprains influenced by mechanical and functional factors. CAI is characterized by the ankle joint’s failure to maintain its position during movement, contributing to talus articular degeneration and an increased risk of osteoarthritis [1]. Balance is a critical skill for controlling human movement and is essential in both static and dynamic positions, particularly for older people or those with chronic health issues such as joint instability. The integration of the visual, proprioceptive, and vestibular systems governs balance. Typically, lower-extremity proprioception is the primary controller. Injury or malfunction prompts compensatory mechanisms, particularly in the trunk and hip areas [2]. A single-leg stance is a common way to induce balance perturbation, challenging the postural control system using a narrow base of support. Therefore, the use of single-leg tests to measure postural stability in clinical and research settings is both logical and warranted. The single-leg stance emphasizes the need for the neuromuscular system to maintain equilibrium. In addition to its functional applicability, assessing postural stability on one leg reduces the number of peripheral sensory sources and muscle strategies available for maintaining balance. This reduction in compensatory mechanisms provides a more focused evaluation of inherent postural control abilities [3].

The quantitative information on dynamic balance assessment was reviewed and summarized in a previous study, which identified the Star Excursion Balance Test (SEBT) as the primary clinical dynamic balance evaluation for individuals with balance deficits, particularly those with ankle instability [4,5]. The SEBT involves reaching in multiple directions while maintaining a single-leg stance, providing quantitative measures of reach distance in different directions. To enhance practicality and reduce complexity, a simplified modified Star Excursion Balance Test (mSEBT) was introduced, focusing on three specific directions: anterior, posteromedial, and posterolateral, with high sensitivity for detecting balance impairment. Furthermore, an mSEBT device was developed for the Y-Balance Test (YBT), a streamlined version of the SEBT that simplifies the assessment [6]. The single-leg hop test is another commonly used quantitative test for assessing functional balance in prior studies. It involves measuring either the distance hopped or the time taken to complete the hop, providing objective data on dynamic balance performance [7]. In addition to these quantitative measures, qualitative data on physiological and biomechanical aspects of balance have been obtained using various advanced methods [8,9,10]. For instance, force plates are used to assess the center of pressure (COP) and provide insights into postural control strategies. Accelerometers are employed to detect the center of mass (COM), offering information on overall body movement and stability. Electromyography (EMG) is used to measure muscle activation patterns, revealing the neuromuscular control involved in maintaining balance. Ultrasound imaging (USI) is used to observe changes in muscle morphology, such as muscle thickness and architecture, contributing to our understanding of musculoskeletal adaptations in response to balance training or deficits.

For the effective translation of research findings into clinical applications and to guide further research in populations with ankle stability deficits, it is crucial to establish precise measurements and outcomes for assessing dynamic balance. This was the primary objective of our scoping review, which aimed to systematically present and synthesize the prevalent tasks and outcomes employed in assessing dynamic balance in individuals with chronic ankle instability. The review also sought to clarify and investigate gaps in existing qualitative studies within these domains, ensuring a comprehensive understanding of both quantitative performance metrics and qualitative physiological and biomechanical characteristics.

## 2. Methods

### 2.1. Protocol and Registration

This scoping review follows the Preferred Reporting Items for Systematic Reviews and Meta-analyses Extension for Scoping Reviews (PRISMA-ScR) guidelines [11]. Prior to data extraction, our review was registered with the Open Science Framework (https://doi.org/10.17605/OSF.IO/FU5CY) on 14 August 2024.

### 2.2. Search Strategy

A scoping review was conducted to elucidate the salient characteristics of the concepts under consideration. Specifically, the focus was on identifying assessments encompassing both tasks and outcome measures pertaining to physiological and biomechanical balance variables among individuals with a history of ankle sprain or CAI. This comprehensive exploration included four databases: PubMed, Scopus, Web of Science, and MEDLINE, with no temporal restrictions imposed, extending until August 2023. The search strategy employed a combination of MeSH terms and predefined keywords, the details of which are presented in Table 1. The criteria for English-language publication were consistently applied throughout both search periods. The initial search was conducted in September 2023 and subsequently updated in April 2024, with additional searches performed up to that date to maintain its relevance and currency.

### 2.3. Study Selection and Eligibility Criteria

Two authors (CP and TJ) conducted the search using the web-based systematic review software ‘Parsifal’ version 2.2 and made selections after duplicates were removed. Following the selection process, the same independent authors screened titles, abstracts, and full texts according to eligibility criteria. In cases where discrepancies in opinion arose, the two authors engaged in discussions to reconcile their differences and reach a consensus. In instances where a conclusive resolution was elusive, the final decision was deferred to the third author (PYL)

The criteria for title and abstract screening encompassed the following parameters: (1) inclusion of adult participants aged 18 years or older; (2) the study targeted assessments at the lower limb; (3) the investigation focused on chronic or functional ankle instability; and (4) the study was an original article. Subsequently, full-text screening involved more specific criteria: (1) the selected studies must incorporate at least one key characteristic representing the balance problem, including accelerometer, force plate, electromyography, ultrasonography, strength, or clinical test; (2) the study must include a dynamic or functional balance test with the biomechanical or physiological variables evaluation; (3) the functional task assessed should be related to single-leg loading; and (4) the absence of other related problems or conditions affecting the lower limbs or back region.

### 2.4. Data Charting and Result Categorization

The two authors (CP and TJ) retrieved data from scoping review records using a data extraction tool they developed. The tool included specific details about the population (age and sample size), inclusion criteria (history of ankle sprain or CAI and severity), and assessments of physiological and biomechanical variables, as shown in the descriptive data extraction of each study. Any disagreements that arose between the authors were resolved through discussion or by consulting a third author (PYL).

### 2.5. Synthesis and Reporting of the Results

The analysis of all included articles involved examining factors indicative of both physiological and biomechanical characteristics. Outcomes were categorized based on participant characteristics, the nature of the tasks undertaken, and the specific outcome measurements employed. Deliberating on the strengths and identified gaps in the available evidence holds practical significance for the shaping of future clinical practice and research.

## 3. Results

### 3.1. Selection of Sources of Evidence

**Figure 1:**
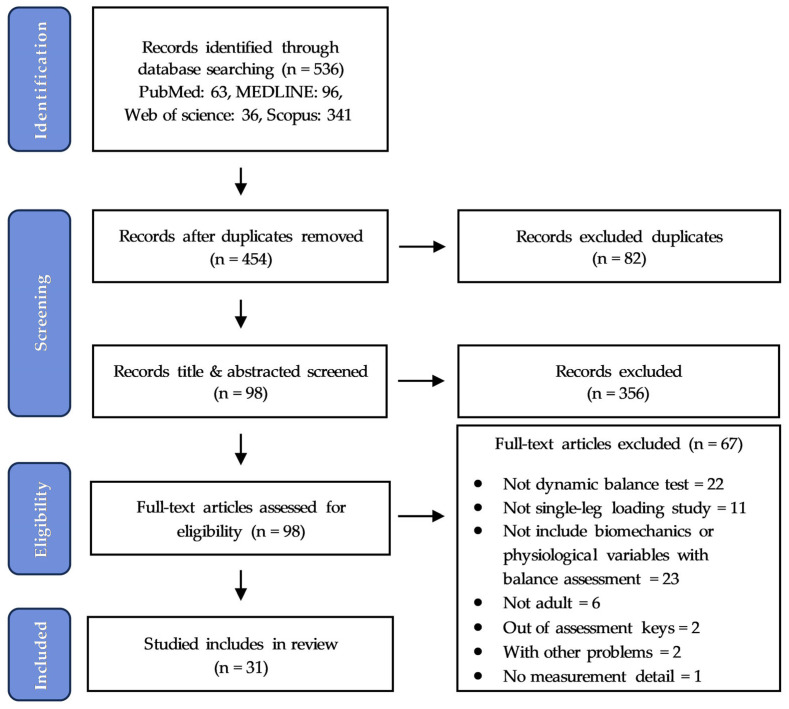
PRISMA-ScR flow diagram of the study selection process.

We identified 536 articles related to ankle issues and dynamic balance assessments. Upon the initial examination, 82 of these were found to be duplicates. After removing the duplicates, we conducted a rigorous screening process to evaluate titles, abstracts, and full texts based on our established criteria. From this pool, 31 studies met our inclusion criteria and were included in our review (Figure 1 and Table S1). These studies were analyzed to gain insight into the conditions related to ankle impairment, participants involved, tasks undertaken, and outcome measures.

The study selection process is illustrated in Figure 1.

### 3.2. Characteristics of Sources of Evidence

Eighteen studies investigated dynamic balance as the primary outcome, including six studies employing the clinical dynamic balance SEBT [12,13,14,15,16,17], using the YBT testing kit [18,19,20,21,22,23] and the Biodex balance system (BBS) [17,24,25,26,27,28]. Furthermore, biomechanical variables were assessed in 11 studies [12,13,14,17,18,22,24,25,26,27,28], and another five focused on physiological evaluation [13,14,18,19,23]. Functional balance outcomes were central in 18 studies [15,16,20,21,29,30,31,32,33,34,35,36,37,38,39,40,41,42], with 17 incorporating biomechanical assessments [15,16,20,21,29,30,31,32,33,34,35,36,37,38,39,40,41] and only four emphasizing physiological outcomes [30,34,37,42]. Additionally, four studies conducted measurements encompassing both dynamic and functional balance domains [15,16,20,21].

### 3.3. Results of Source of Evidence

**Figure 2:**
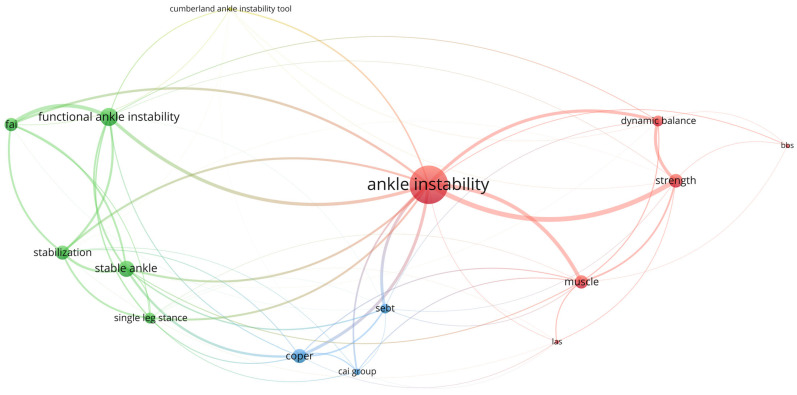
Visualization of keyword co-occurrence network: total link strength analysis.

Extracted data are presented in Table S1. Figure 2 was generated by the VOSviewer version 1.6.20 (CWTS, Leiden University, Leiden, The Netherlands) using the Total Link Strength metric, representing the cumulative strength of a particular keyword’s co-occurrence with other keywords across the included articles. Keywords with higher Total Link Strength values are displayed with larger labels, indicating greater importance in the network. The thickness of the links between keywords reflects the strength or frequency of their connections, with thicker links indicating stronger or more frequent associations. As depicted in Figure 2, ankle instability emerges as the primary association, particularly functional ankle instability (FAI), which is influenced by alterations in both dynamic balance and muscle strength. Furthermore, the figure depicts a connection between patients with CAI and FAI following injury, with the evaluation including both single-leg dynamic balance and self-reporting ankle instability questionnaires. The SEBT can serve as the primary dynamic balance test for the ankle instability population, as it correlates with muscle strength and ankle stability. Additionally, the instability questionnaire score is impacted by the level of dynamic balance and strength.

### 3.4. Synthesis of Results

#### 3.4.1. Inclusion Criteria

Table 2 displays the criteria set by the International Ankle Consortium for categorizing participants with CAI in this study. Among these 31 studies, a history of at least one ankle sprain was a prominent inclusion criterion in 28 studies (90%). These criteria encompass the onset period, accompanying symptoms, and physical limitations. Next, the sensation of the ankle “giving way” was reported in 25 of the studies (81%). Meanwhile, only 17 (46%) and 5 (22%) studies incorporated self-administered questionnaires to assess instability and functional capabilities, respectively.

#### 3.4.2. Tasks

The tasks were categorized into two distinct groups: those pertaining to dynamic balance and those to functional balance assessment (Table 3). Within the realm of dynamic balance studies, the assessment tool used was the SEBT (67% of 18 studies), in which the anterior reach direction was the most frequently evaluated aspect. The remaining studies employed the BBS in conjunction with Power Plate equipment, constituting 33% of the total studies. In the context of functional balance studies, the most prevalent tasks included single-leg jumps and landings, featured in 44% of the 18 studies. Subsequently, the drop landing task was investigated in 39% of the studies.

#### 3.4.3. Outcome Measurements

In the evaluation of clinical dynamic balance, a diverse array of outcome measures was employed. The predominant approach involved using the normalized reach distance score as quantitative evaluation, which was used in 11 studies, representing 92% of the 12 studies reviewed. Alternatively, one study employed the mean value. In terms of qualitative measurement, a notable portion of the studies (44% of 17 studies) emphasized stability parameters, such as the stability index (SI) and the activity of the COP, using power plates and force plates. Concurrently, five studies employed motion cameras and accelerometers to examine kinematic aspects. Regarding physiological parameters, electromyography emerged as the predominant parameter in the muscle anthropology domain (19% of 17 studies). Furthermore, only one study addressed muscle morphology during balance control.

**Figure 3:**
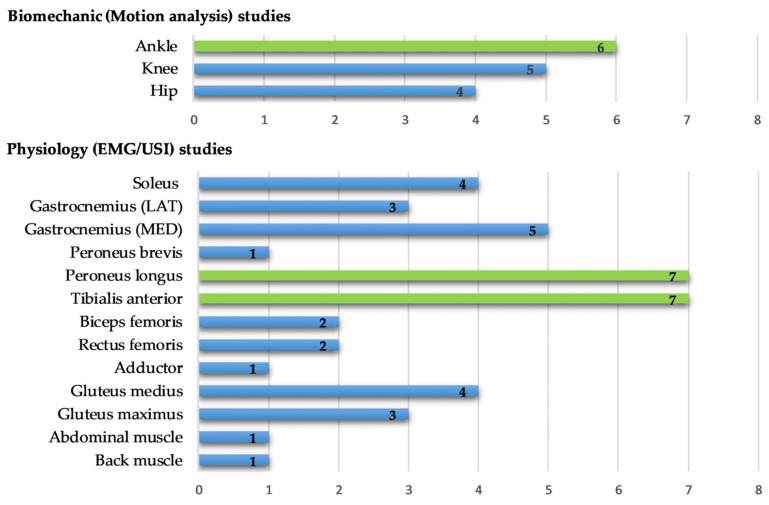
Frequency of target structure for biomechanical and physiological studies for balance evaluation.

Most clinical function task studies require laboratory equipment, including force plates, video cameras, and EMG for data collection. The quantification of kinetic variables involved the use of force plates. Among these studies, seven (44% of 16) calculated the COP displacement, while six (38% of 16) specifically identified the time to stabilize (TTS) and four (25% of 16) used SI as a significant outcome. Kinematic variables were predominantly assessed through a range of motion (ROM) using video motion analysis and electromagnetic fields. Additional kinetic and kinematic parameters related to ankle dynamic balance measurements are detailed in Table 3. In the realm of EMG studies, five articles addressed muscle activation during single-leg loading control, employing the integration of EMG signals in 60% of the five studies, while other studies used maximum voluntary contraction (MVIC/MVC), latency, and co-contraction index. Additionally, among EMG and USI studies in the CAI population, the peroneus longus and tibialis anterior muscles were the most frequently examined, and the ankle joint was the primary focus for analyzing joint angles during balance assessments (Figure 3).

Muscle strength evaluations commonly relied on MVC, while proprioceptive testing predominantly employed the degree of position error. Additionally, one study used the basic goniometer as the tool for ROM evaluation.

## 4. Discussion

### 4.1. Summary of Evidence

#### 4.1.1. Inclusion Criteria

The inclusion criteria were critical for selecting relevant participants and directly affected the study’s objectives and outcomes. This scoping review focuses on chronic ankle stability and uses these criteria as a foundation for the analysis. The findings of this review have the potential to inform future research in this field. We delineated the primary criteria across four key issues to ensure a comprehensive and targeted approach.

First, a history of ankle sprain, particularly a lateral ankle sprain, is the primary cause of ankle instability, with the severity and frequency of injuries affecting the degree of structural deterioration. Most studies adhere to guidelines requiring at least one major ankle sprain and a minimum of one day of disrupted physical activity as a primary criterion [43], although descriptions such as lateral, acute, and significant ankle sprains are commonly used; they essentially refer to the same condition. The standard definition used is “at least one (acute, significant, or lateral) ankle sprain”, with the initial injury typically occurring within 6 months to 5 years, although 12 months is the most common duration per the CAI Consortium guidelines. Injury severity is a key predictor of future symptoms, and some studies have used severity levels to classify injuries [15,38,44]. A common trend in these studies was to use symptom duration, often 1 to 3 days, as a criterion for assessing injury severity.

Second, the sensation of giving way, instability, or recurrent ankle sprain is a crucial factor for CAI. This sensation is defined as frequent, uncontrolled episodes of excessive inversion of the rear foot without an acute lateral ankle sprain [43]. Most studies identify this as one to three episodes of “giving way” during physical activities within a span of 6 to 12 months, indicating chronic instability as a lasting consequence of the initial injury.

Third, assessing instability levels often involves self-reported measures, particularly the Ankle Instability Instrument (AII) and the Cumberland Ankle Instability Tool (CAIT). AII and CAIT focus on post-acute injury history and ongoing perception of instability, with excellent test-retest reliability at 0.95 and 0.99, respectively. Additionally, the CAIT demonstrated 82.9% sensitivity and 74.7% specificity, with a discrimination score of 27.5 [45]. However, the cutoff score was adjusted to 25 to improve sensitivity and specificity in the CAI group [46]. Cross-cultural studies validated the effectiveness and reliability of the revised cutoff score [47,48].

Finally, a functional questionnaire, predominantly comprising the Foot and Ankle Ability Measures in Activities of Daily Living (FAAM-ADL) and Sports Function (FAAM-S), emerged as the last criterion. The ADL subscale of the FAAM addresses functional limitations in daily activities, while the sports subscale pertains to sports-specific skills. Both subscales demonstrate commendable personal and item reliability, with values of 0.87 and 0.99 for the ADL subscale and 0.89 and 1 for the sports subscale. Moreover, the validity of these measures provided good evidence [49]. The consortium recommends scores below 90% for the FAAM-ADL and below 80% for the FAAM-S as indicators of chronic ankle instability [43].

Some studies have incorporated clinical criteria, such as the ankle anterior drawer test and talar tilt test [29,31,38,42]. Additionally, participants categorized as “copers”, mirroring CAI criteria but not giving way sensations [13,14,29,33,39], exhibit higher scores in self-reported questionnaires, surpassing 25 in CAIT and achieving full scores in both FAAM subscales [33].

#### 4.1.2. Tasks and Outcome Measurement

This scoping review demonstrates the tasks and outcome measures involved in dynamic and functional balance tests across clinical and equipment-based approaches. Additionally, it addresses pertinent factors related to balance management.

#### 4.1.3. Clinical Dynamic Balance Test

The SEBT is a prominent tool for assessing clinical dynamic balance. SEBT, with its eight designated directions, has been widely utilized in chronic ankle instability studies, emphasizing a single-leg loading paradigm with leg reach. Notably, the anterior (ANT), posteromedial (PM), and posterolateral (PL) directions have gained significance in research, aligning with the newly updated practical guidelines [4], which relate to the chosen direction for the developed YBT assessment tool. Moreover, the PM direction was the most representative of the overall direction score in the limbs with and without CAI [50] and contributed the most to the CAIT score [51]. During the test, the participants were instructed to stand on the testing leg with their hands on their hips and extend the other leg as far as possible in the designated direction. Subsequently, when the most distal part of the reaching foot contacted the floor, the participant returned to the initial position while maintaining equilibrium. The test involved four practice trials, followed by three testing trials in each direction, with four practice trials as the optimal learning curve consideration and fatigue mitigation [4,5]. Failed tasks were defined as instances of excessive sway, both feet in contact with the ground, hands off the hips, and an inability to return to the initial position. Recent systematic reviews have confirmed the reliability of this method in healthy adults. The interrater reliability, as indicated by the Intraclass Correlation Coefficient (ICC), was noteworthy, with values of 0.88, 0.87, and 0.88 for the ANT, PM, and PL directions, respectively. Similarly, intra-rater reliability demonstrated strong consistency for the ANT, PM, and PL directions, with ICC values of 0.88, 0.88, and 0.90, respectively [52], further supporting their utility in assessing dynamic balance. The outcome measurement was derived from the average of three reach trials in each direction and reported as individual and overall scores, which are usually normalized by leg length.

#### 4.1.4. Equipment-Dependent Dynamic Balance Test

In dynamic balance studies, the primary quantitative metric is often the maximum reach distance; however, a comprehensive understanding involves qualitative factors such as kinetic and kinematic variables [12,13,14,18,19,22,23]. An investigation of kinematic variables used 3D motion analysis to uncover intricate details of the hip, knee, and ankle ROM, revealing decreased knee flexion compared to healthy individuals. Despite the lack of a significant correlation between the maximum reach distance and the sagittal plane ROM of the lower limb in patients with CAI [12,18], CAI has been proven to induce trunk compensation in both the sagittal and coronal planes [2]. Another kinematic parameter study was the COM, which was analyzed using accelerometers, including jerk, root mean square (RMS) of sway, and mean velocity, as the primary outcomes correlate positively with clinical dynamic balance tests, particularly in the posteromedial direction [22]. This result emphasizes the importance of proximal compensation after ankle stability deficits.

In addition, the ground reaction force and muscle activation are commonly used. The ground reaction force measured by force plates was employed to analyze the COP patterns in studies of dynamic balance control. Metrics such as sway velocity, ellipse area, and path length were examined, revealing the significant effects of CAI on COP control parameters, particularly sway velocity, during single-leg tasks across various directions [13]. Furthermore, the isokinetic protocol for dynamic balance assessment, referred to as BBS, was extensively employed to calculate the SI. The BBS demonstrated reliability as a tool to determine the overall percentage and SI parameters, with ICC values of 0.83 and 0.75, respectively. However, it failed to exhibit a correlation with functional performance measurement, likely due to the distinct aspects of balance control being evaluated [53].

Muscle activation, assessed through EMG during dynamic balance control (SEBT), indicates decreased MVC of the tibialis anterior and gluteus maximus, with increased activation of the peroneus longus and medial gastrocnemius, depending on the direction [13,14]. However, muscle activation alone may not fully represent neuromuscular control during functional activities because of diverse muscle characteristic factors. USI has been used to investigate muscle quality during dynamic balance control to explore other physiological variables. These studies revealed the functional activation ratio (FAR) and preferential activation ratio as neuromuscular control metrics. The FAR was calculated by dividing the average muscle thickness during a task by the average quiet-stance muscle thickness. Moreover, there was a notable trend indicating an improvement in hip neuromuscular control during single-leg loading in individuals with CAI [18]. Nevertheless, few USI studies in static positions and isometric contractions have revealed changes in muscle morphology characteristics, including hypertrophy and overactivity in synergist muscles, in contrast to less activity and hypotrophy in primary stabilizer muscles [54].

#### 4.1.5. Clinical Functional Balance Test

The functional balance test includes the hop, drop landing, and jump-and-landing tests. However, this review did not uncover clinical hop tests incorporating biomechanical and physiological measurements. Nonetheless, previous studies have used original clinical hop tests such as the single-hop test, triple-hop test, timed hop test (6 m hop), and crossover hop test [7,55], with the forward single hop being the most commonly used functional stability test. The hop test was implemented as the first objective to assess knee control following a knee injury. A recent study established a correlation between the single-hop test and clinical dynamic balance as evaluated by the YBT, with a specific focus on ankle joint balance control. This finding suggests that the single-hop test can effectively evaluate functional balance deficits in patients with ankle impairment [56], with good reliability (ICC = 0.96, 0.95, 0.66, and 0.96, respectively) [57]. Additionally, various studies have introduced hop tests for ankle deficits, including the figure-of-eight, side-hop, 6 m crossover, square, and multiple hop tests [58,59,60]. Ankle function tests aim to enhance diagonal and mediolateral control by recognizing the challenges of controlling ankle joint arthrokinematics and mechanical insufficiency during movement in these directions. The outcome measurements of these tests are typically reported as quantitative parameters such as distance, time taken, and the number of errors made.

#### 4.1.6. Equipment-Dependent Functional Balance Test

The equipment-dependent functional balance test focused on qualitative data collected during the landing phase in both drop and jump landings, employing force plates to describe the kinetic variables [15,16,20,21,29,31,32,33,36,38,39,40,41]. Force plates are considered the gold standard for balance assessment, particularly in a margin of stability evaluation, which is investigated through COP movement to counteract shifts during functional tasks and adjust the body’s position to ensure that the COM remains within a stable range relative to the base of support [61]. Several recent studies have utilized COP displacement in the AP and ML as the primary outcome, followed by TTS, SI, and COP areas. COP displacement and area reflect body weight shift through the amplitude of COP movement. Conversely, the stability indices, including the vertical stability index, anteroposterior stability index and mediolateral stability index, measure the standard deviation fluctuations around a zero point. The resulting dynamic postural stability index was normalized by the participant’s body mass using a formula incorporating the ground reaction force in three axes [39]. TTS measures the duration from foot contact to the midpoint of stable joint conditions in both the AP and ML directions, with TTS in the AP direction being the most empirically validated parameter for landing tests [9]. In landing tests, a shorter TTS often indicates better proprioceptive and neuromuscular responses, thereby reducing the risk of injury associated with unstable landings.

Video cameras and electromagnetic fields were used in several studies [21,29,31] to analyze kinematic variables similar to those used in the dynamic balance task. Specifically, the ROM of the hip, knee, and ankle during the initial landing and maximum knee flexion phases has been reported.

Physiological evaluation, facilitated by EMG, was integral to this study, allowing for the observation of ankle muscle activation during the landing phase. Emphasis was placed on the latency of the peroneus longus and brevis after landing, revealing a significant delay in the latency of the peroneus longus [37]. This outcome, which is consistent with recent research, indicates that ankle sprains contribute to a diminished reaction time of the peroneus longus and ankle neuromuscular control, resulting in a reduction in dynamic balance and performance and an increased risk of recurrent injuries [51,62]. However, the available information regarding the correlation between muscle activation delay and the dynamic control capacity of the ankle in both dynamic and functional tasks remain limited.

#### 4.1.7. Muscle Strength

Muscle strength is a crucial facet of physical fitness that regulates dynamic balance. This connection arises from muscle activation and isometric contractions that are initiated in response to balance perturbations. Recent investigations have frequently employed isokinetic equipment for assessing ankle muscle strength owing to its recognized status as the gold standard, offering high reliability in isometric strength measurement across various bodily movements. Within the context of CAI, the peak torque of the ankle stabilizer muscles is often assessed, followed by the MVIC of the hip abductor, using a hand-held dynamometer [28,63,64], which plays a primary role in single-leg standing. While previous reviews have corroborated the present findings, they have also underscored the limitations associated with the use of isokinetic dynamometers. They determined that the isokinetic dynamometer is costly equipment that only allows for bi-planar motions of plantar flexion/dorsiflexion and inversion/eversion. Therefore, the intricate tri-planar motions of the ankle joint cannot be measured properly using isokinetic devices. Furthermore, the isometric strength indicates static rather than dynamic control [65]. However, hand-held dynamometers are susceptible to the influence of tester strength, potentially compromising measurement accuracy [65].

#### 4.1.8. Limitations

We acknowledge the limitations of our review. We primarily sourced publications from a limited number of publicly available databases. It would be beneficial to include additional sport-specific resources to enhance the relevance of our review across various sports disciplines. Additionally, our scoping review did not explore other factors influencing the decision-making process for selecting specific balance tests in each sports category, which could elucidate the rationale behind these test choices. Nonetheless, the inclusion of such studies would further underscore our findings and conclusions.

#### 4.1.9. Further Research

This scoping review emphasizes the ongoing need for developing and applying standardized outcome measures to evaluate the dynamic balance of the CAI population. Based on our findings, a qualitative study on dynamic balance control is necessary. More evidence is needed on the functional neuromuscular control of ankle stabilizer muscles during various activities. Addressing this knowledge gap requires a comprehensive description of muscle characteristic adaptations across dynamic tasks, along with a focus on the COM of the entire body and joint displacement in the context of dynamic balance. In addition, there is a critical need to elucidate isotonic strength measurement methods to assess dynamic ankle strength effectively. Consequently, future research should refine and implement these methodologies to enhance our understanding of the dynamic balance within the CAI population.

## 5. Conclusions

This scoping review provides a thorough analysis of the methods used to monitor physiological and biomechanical variables in individuals with CAI. This highlights the prevalent use of the SEBT, particularly in the anterior, posteromedial, and posterolateral directions, with the normalized reach distance as a common clinical outcome measure. Functional balance is often assessed by using landing tasks and COP displacement as biomechanical variables. EMG analysis of the tibialis anterior and peroneus longus muscles using %MVIC/MVC and integration of the EMG signal are key methods for examining physiological aspects. Despite some variability in the study inclusion criteria, there was a consistent focus on historical data, sensations, reports of instability, and functional limitations. However, significant gaps exist in the knowledge regarding COM dynamics and muscle architecture. Specifically, the precise role and adaptation of COM dynamics in maintaining balance in individuals with CAI remain underexplored. Similarly, detailed insights into muscle architecture changes, assessed through advanced imaging techniques, are limited. Addressing these gaps is crucial for a comprehensive understanding of the underlying mechanisms of balance deficits and the development of targeted interventions for CAI.

## Figures and Tables

**Table 1:** Search parameters.

Descriptor	Search Item (MeSH/“Keyword”)
Target	Ankle, Muscle/ “muscle”, “muscular”, “ankle”
Impairments	Joint instability, Sprains and Strains/ “ankle instability”, “chronic ankle instability”, “functional ankle instability”
Condition	Weight-Bearing/ “single-leg stance”, “single-leg squat”
Balance parameter terms	Psychomotor performance, Postural balance, Biomechanic, Electromyography, Diagnostic Imaging, Muscle Strength/ “balance”, “dynamic balance”, “functional balance”, “accelerometer”, “center of mass”, “clinical test”, “field test”, “ground reaction force”, “center of pressure”, “muscle activation”, “electromyography”, “ultrasound image”, “ultrasonography”, “inertial measurement unit”, “muscle strength”

**Table 2:** Description of inclusion criteria for CAI and FAI of 31 included articles.

Inclusion Criteria	Number of Articles (%)	Most terms Used in Articles	Number of Articles (%)
A history of at least one significant ankle sprain. The initial sprain must have occurred at least 12 months before study enrollment associated with inflammatory symptoms (pain, swelling, etc.) Resulted in at least one interrupted day of desired physical activity.	28 (90)	“at least 1 (acute, significant, lateral, substantial) ankle sprain”	16 (52)
A history of the previously injured ankle joint “giving way” and/or recurrent sprain and/or “feelings of instability”.	25 (81)	“at least or 2 episodes of giving way”	10 (32)
Self-reported ankle instability should be confirmed with a validated ankle instability-specific questionnaire using the associated cutoff score.	17 (55)	“CAIT ≤ 24”	5 (16)
A general self-reported foot and ankle function questionnaire is recommended to describe the level of disability of the cohort.	5 (16)	FAAM-ADL < 90%, FAAM-Sport < 80%	3 (10)

Abbreviation: CAIT, Cumberland Ankle Instability Tool, FAAM-ADL, Foot and Ankle Ability Measure—Activities of Daily Living; FAAM-Sport, Foot and Ankle Ability Measure—Sport.

**Table 3:** Frequency of task and outcome measurement used in balance control and related assessment.

	Tasks	Number (%)	Outcome Measurement		Number (%)
			Quantitative Parameters	Qualitative Parameters	
Dynamic balance [12,13,14,15,16,17,18,19,20,21,22,23,24,25,26,27,28] (*n* = 17)	Total = 18		Total = 12	Total = 17	
	SEBT/mSEBT	6 (33)	Mean value reach distance		1 (8)
	Anterior	6 (100)	Normalized reach		
	Anteromedial	1 (17)	distance score		11 (92)
	Medial	3 (50)		Power plate	6 (38)
	Posteromedial	4 (67)		Stability index	6 (100)
	Posterior	2 (33)		Force plate	1 (6)
	Posterolateral	4 (67)		COP area	1 (100)
	Lateral	1 (17)		COP velocity	1 (100)
	Composite	1 (17)		COP displacement	1 (100)
	YBT	6 (33)		2D/3D motion analysis	3 (19)
	BBS	6 (33)		ROM	2 (67)
				Reach length	1 (33)
				Accelerometer	2 (13)
				Magnitude of acceleration	1 (50)
				RMS sway	1 (50)
				COM Velocity	1 (50)
				COM jerkiness	1 (50)
				EMG	4 (19)
				%MVIC/MVC	3 (100)
				Onset time	1 (33)
				Normalize mean	
				amplitude	1 (33)
				USI	1 (6)
				FAR	1 (100)
				Activation ratio	1 (100)
Functional balance [15,16,20,21,29,30,31,32,33,34,35,36,37,38,39,40,41,42] (*n* = 18)	Total = 18			Total = 25	
	SLDL	7 (39)		Force plate	16 (64)
	Forward	4 (50)		COP area	3 (19)
	Lateral	2 (25)		COP velocity	1 (6)
	Medial	2 (25)		COP displacement	7 (44)
	SLJL/SLHS/VJT	8 (44)		Ground reaction force	2 (13)
	Vertical	4 (50)		Stability index	4 (25)
	Forward	3 (37)		Time to stabilize	6 (38)
	Diagonal	1 (13)		Time to boundary	1 (6)
	SLS with kicking	3 (17)		Time to peak	1 (6)
	task			2D/3D motion analysis	3 (12)
				ROM	3 (100)
				Velocity	1 (33)
				Time to maximum	1 (33)
				Electromagnetic field	1 (4)
				ROM	1 (100)
				Joint translation	1 (100)
				EMG	5 (20)
				Integrate signal	3 (60)
				%MVIC/MVC	1 (20)
				Latency	1 (20)
				Co-contraction index	1 (20)
Related	Total = 4			Strength	
assessment	Isokinetic			MVC	1
[15,23,28,42]	dynamometer	2 (50)		Proprioception	
(*n* = 4)	Goniometer	2 (50)		Joint position sense	2
				(degree of positioning error)	
				ROM	1

Abbreviation: SEBT: Star Excursion Balance Test; mSEBT: Modified Star Excursion Balance Test; YBT: Y-Balance Test; BBS: Berg Balance Scale; COP: Center of Pressure; ROM: Range of Motion; RMS: Root Mean Square; COM: Center of Mass; EMG: Electromyography; %MVIC/MVC: Percentage of Maximum Voluntary Isometric Contraction/Maximum Voluntary Contraction; USI: Ultrasound Imaging; FAR: Functional Activation Ratio; SLDL: Single-Leg Drop Landing; SLHS: Single-Leg Hop Stabilization; SLJL: Single-Leg Jump and Landing; SLS: Single-Leg Stance; VJT: Vertical-Jump Test.

## Data Availability

All data generated or analyzed during this study are included in this published article.

## Supplementary Materials

The following supporting information can be downloaded at: https://www.mdpi.com/article/10.3390/sports12080224/s1, Table S1 Author, inclusion criteria, participant’s characteristics, and tasks and outcome measures in included studies.

## List of Abbreviations

ADL	activities of daily life
AII	ankle instability instrument
AJFAT	ankle joint functional assessment tool
ANT	anterior
CAI	chronic ankle instability
CAIT	Cumberland ankle instability tool
COM	center of mass
COP	center of pressure
EMG	electromyography
FAAM	foot and ankle ability measure
FAI	functional ankle instability
FAR	functional activation ratio
IdFAI	Identification of Functional Ankle Instability
mSEBT	modified star excursion balance test
MVC	maximum voluntary contraction
MVIC	maximum voluntary isometric contraction
PM	posteromedial
PL	posterolateral
RMS	root mean square
ROM	range of motion
SEBT	star excursion balance test
SI	stability index
SLDL	single-leg drop landing
SLHS	single-leg hop stabilization
SLJL	single-leg jump and landing
SLS	single-leg stance
SLST	single-leg squat
TTS	time to stabilize
USI	ultrasound imaging
VJT	vertical jump test
YBT	Y-balance test
