# Supplementary material for: Biomechanical and Physiological Variables in Dynamic and Functional Balance Control during Single-Leg Loading in Individuals with Chronic Ankle Instability: A Scoping Review
Source: Sports (Basel). 2024 Aug 16;12(8):224. doi: 10.3390/sports12080224 (PMC11359178; doi:10.3390/sports12080224)
Supplement: Supplementary file 1 [file sports-12-00224-s001.zip › sports-3127697-supplementary.pdf]

Table S1 Author, inclusion criteria, participant's characteristics, and tasks and outcome measures in included studies

Author (Y)	Inclusion criteria	Participants	Tasks and outcome measures
**Abdo (2020)** [24]	(1) age ranging between 18 and 35 years old; (2) CAIT <27; (3) ≥ 1 recurrent sprain within the past year.	N: 65<br>Experiment (CAI): 31<br>Age (median): 22 yrs.<br>Control (Healthy): 34<br>Age (median): 22 yrs.	BBS: dynamic balance protocol<br>Power plate(BBS): overall stability index<br>Accelerometer: magnitude of acceleration in all axes for calculating overall stability index
**Asimenia (2013)** [25]	(1) ≥ 1 repeated injury or perception of ankle instability or "giving away" in the unstable ankle; (2) no evidence of mechanical instability using anterior drawer test; and (3) pain-free, full weight bearing, and normal gait at the time of study.	N: 30<br>Age: 20.58±0.64 yrs.	BBS: dynamic balance protocol<br>Power plate(BBS): overall stability index, anteroposterior stability index, mediolateral stability index
**Brown (2008)** [29]	All group<br>(1) history of acute inversion ankle sprain requiring immobilization or non-weight bearing for at least 3 days.<br>MAI group<br>(1) 2 episodes of ''giving way" in the past 12 months; (2) (+) anterior drawer and/or talar tilt test<br>FAI group	N: 63<br>Experiment (MAI): 21<br>Age: 22.38±4.30 yrs.<br>Experiment (FAI): 21<br>Age: 22.14±3.83 yrs.<br>Experiment (Coper): 21<br>Age: 21.71±4.85 yrs.	SLDL: anterior step off from box height 32 cm<br>Force plate: peak normalized ground reaction force, time to peak ground reaction force in V/A/P/M/L direction<br>Electromagnetic tracking device: ROM and translation of ankle and knee joint at initial contact and maximum contact

Author (Y)	Inclusion criteria	Participants	Tasks and outcome measures
	(1) 2 episodes of ‘‘giving way” in the past 12 months; (2) (-) anterior drawer and/or talar tilt test.<br>Coper group<br>(1) no repeated episodes of ‘‘giving way” and no ankle sprains in the past 12 months.		
**Conceição (2016)** [30]	(1) history of ≥ 2 ankle sprains, with at least 1 sprain within the 6 months before the study; (2) a sensation of ankle instability (giving way); (3) CAIT < 28	N: 44<br>Experiment: 22<br>Age: 24±4 yrs.<br>Control: 22<br>Age: 22±3 yrs.	SLS: kicking a ball by the other leg<br>EMG: normalize integrated signals of soleus, medial gastrocnemius, lateral gastrocnemius, tibialis anterior, peroneus longus, biceps femoris, rectus femoris, and sum of Integrated signals<br>Force plate: COP displacement (AP/ML), COP area
**DeJong (2020)** [18]	(1) ≥ 1 substantial ankle sprain in 12 months; (2) IdFAI > 11, FAAM < 90%, FAAM-sport < 80%, and CAIT < 24	N: 40<br>Experiment (CAI): 20<br>Age: 21.7±2.32 yrs.<br>Control (Healthy): 20<br>Age: 21.2±2.79 yrs.	YBT: %normalized reach distance score<br>Ultrasound imaging: FAR of Gmax and Gmed, preferential-activation ratio of Gmax and Gmed<br>3D motion analysis: ROM of hip flexion, knee flexion, and ankle dorsiflexion
**Elsotohy (2021)** [26]	(1) history ≥ 1 ankle sprain, with the initial sprain occurring more than 1 year before the study; (2) ≥ 2 episodes of ‘‘giving way’’ in the last 6 months	N: 32<br>Age: 20.96±1.69 yrs.	BBS: dynamic balance protocol

Author (Y)	Inclusion criteria	Participants	Tasks and outcome measures
	before the study; (3) ≥ 2 ‘‘yes’’ answers on the questions (4 to 8) of MAII.		Power plate(BBS): overall stability index, anteroposterior stability index, mediolateral stability index
**Fatima (2020)** [19]	(1) a history of ≥ 2 ankle inversion injuries in the same ankle for the past 2 years, which had required a period of protected weight bearing or immobilization; (2) a complaint of giving way of the ankle during functional activity	N: 34<br>Experiment (CAI): 17<br>Age: 24.4±2.03 yrs.<br>Control (Healthy): 17<br>Age: 24.6±2.57 yrs.	SLST (5s): in 5 conditions; 1) squat with an anterior reach of YBT, 2) squat with a posteromedial reach of YBT, 3) squat with a posterolateral reach of YBT, 4) squat without Swiss ball, 5) squat with Swiss ball<br>EMG: %MVIC of Gmax and Gmed
**Gottlieb (2024)** [16]	(1) history of ≥ 1 significant ankle sprain that occurred at least 12 months before the study; (2) history of ≥ 2 episodes of “giving way” in the past six months; (3) CAIT ≤ 24 and IdFAI ≥ 11; (4) ability to bear full weight on the injured lower extremity; (5) No skin lesion	N: 29<br>Experiment 1: 14<br>Age: 23.33±1.98 yrs.<br>Experiment 2: 15<br>Age: 24.5±2.23 yrs.	mSEBT: %normalized reach distance score<br>SLJL: forward single-leg drop-jump<br>Force plate: TTS
**Gribble (2004)** [12]	(1) history of ≥ 1 acute ankle sprain that resulted in swelling, pain, and temporary loss of function; (2) a history of multiple episodes of the ankle ‘‘giving way’’ in the past 6 months	N: 30<br>Experiment (CAI): 14<br>Age: 21.9±2.9 yrs.<br>Control (Healthy): 16<br>Age: 22.5±2.4 yrs.	SEBT (ANT/MED/POS): %normalized reach distance score<br>Kinematic analysis (Digital video camera): ROM of hip flexion/extension, knee flexion/extension, and

Author (Y)	Inclusion criteria	Participants	Tasks and outcome measures
			ankle dorsiflexion/plantarflexion at the maximum reach position
**Huang (2014)** [31]	(1) ≥ 1 acute ankle inversion sprain that resulted in swelling, pain, and dysfunction; (2) recurrent ankle sprains or ankle ‘‘giving way’’ in the past 12 months; (3) CAIT < 24; (4) clinically negative anterior drawer and talar tilt tests	N: 30<br>Experiment 1: 10<br>Age: 23.2±2.82 yrs.<br>Experiment 2: 10<br>Age: 23.8±4.13 yrs.<br>Control: 10<br>Age: 23.5±3 yrs.	SLDL: medial and lateral single-leg drop landing from a 16-cm-high platform<br>Force plate: time to stabilize, maximum relative displacement between COM and COP<br>3D motion analysis: hip maximum flexion, extension, internal rotation, external rotation, knee maximum flexion, extension, and ankle in all directions
**Huang (2021)** [42]	(1) participate regularly in sports activities; (2) ≥ 1 prior ankle inversion sprain that results in swelling, pain, and dysfunction over the past 12 months; (3) have experienced multiple ankle sprains or ankle “giving way” events over the past 12 months; (4) CAIT < 24; (5) clinically test negative in anterior drawer and talar tilt tests	N: 30<br>Experiment 1: 10<br>Age: 23.2±2.82 yrs.<br>Experiment 2: 10<br>Age: 23.80±4.13 yrs.<br>Control: 10<br>Age: 23.5±3 yrs.	SLDL: medial single-leg drop landing<br>EMG: Integrated EMG value of tibialis anterior, peroneus longus, medial and lateral head of gastrocnemius and soleus<br>Electrogoniometer: joint position sense; degree of positioning error
**Jaber (2018)** [13]	(1) had a history of ≥ 1 significant lateral ankle sprain that resulted in pain and loss of function of >	N: 48<br>Experiment (CAI): 16	SEBT (ANT/PM/PL): %normalized reach distance score#

Author (Y)	Inclusion criteria	Participants	Tasks and outcome measures
	1 day(for CAI and coper groups); (2) sprain occurred not less than 12 months ago with no complaint of giving way episodes (for copers); (3) had a history of ≥ 2 episodes of 'giving way' in the past 6 months (for CAI group); (4) had no history of ankle sprains (for the control group)	Age: 29.6±4.2 yrs.<br>Experiment (Coper): 16<br>Age: 27.8±4.4 yrs.<br>Control (Healthy): 16<br>Age: 25.8±4.4 yrs.	Force plate: sway velocity, 95% Confidence Ellipse Area, and path length<br>EMG: %MVC, %MVIC of tibialis anterior, peroneus longus, Gmax, Gmed and mean onset time in second of Gmax and Gmed
**Khalili (2022)** [27]	(1) collegiate athletes; (2) history of ≥ 1 significant ankle sprain that required rest and immobility; (3) feeling of ankle instability and "giving way" during daily routine and sports activities; (4) AJFAT score ≥ 26; (5) fully able to tolerate body weight and walk normally	N: 24<br>Experiment 1: 12<br>Age: 24.8±2.6 yrs.<br>Control: 12<br>Age: 25.6±2.5 yrs.	BBS: dynamic balance protocol<br>Power plate(BBS): Overall stability index
**Kwon (2023)** [14]	CAI group<br>(1) history of ≥ 1 significant ankle sprain which involved pain or swelling ≥ 1 interrupted day of desired physical activity and initial ankle sprain occurred ≥ 1 year; (2) ≥ 2 episodes of the ankle giving way within the past 6 months<br>Coper group	N: 60<br>Experiment (CAI): 20<br>Age: 20±2 yrs.<br>Experiment (Coper): 20<br>Age: 23±2 yrs.<br>Control (Healthy): 20<br>Age: 22±3 yrs.	SEBT (ANT/PM/PL): %normalized reach distance score<br>EMG: normalized mean amplitude of tibialis anterior, peroneus longus, and medial head of gastrocnemius.<br>3D motion analysis: detect the distance of the SEBT

Author (Y)	Inclusion criteria	Participants	Tasks and outcome measures
	(1) history of a lateral ankle sprain that required immobilization and/or non-weight bearing for ≥ 3 days; (2) no episodes of the ankle giving way and/or feeling of instability for at least 12 months.		
**Lee (2020)** [21]	(1) AII > 5	N: 14<br>Experiment: 7<br>Age: 22±1.73 yrs.<br>Control: 7<br>Age: 23.57±1.62 yrs.	YBT: %normalized reach distance score<br>SLDL: anterior single-leg drop landing from a 45-cm-high box<br>Force plate: COP amplitude, vertical ground reaction force at initial contact, and maximum knee flexion<br>3D motion analysis: ROM of hip, knee, ankle at initial contact and maximum knee flexion
**Lee (2022)** [20]	(1) a history of ≥ 1 significant ankle sprain occurring > 3 months; (2) a history of 2 giving way episodes within the past 6 months; (3) FAAM-ADL < 90%, FAAM-S < 80%, and AII > 5	N: 28<br>Experiment: 14<br>Age: 21±3 yrs.<br>Control: 14<br>Age: 22±2 yrs.	YBT (EO/SV): %normalized reach distance score<br>SLHS (EO/SV): vertical jump at 50% of maximum jump height<br>Force plate: anteroposterior stability index, mediolateral stability index, vertical stability index, dynamic postural stability index

Author (Y)	Inclusion criteria	Participants	Tasks and outcome measures
**Mahmoud (2023)** [28]	(1) history of a unilateral lateral ankle sprain that occurred within the preceding year before study enrollment,; (2) history of giving way and/or experiences of instability of the affected ankle during daily activities for at least 6 months; (3) CAIT ≤ 24	N: 39<br>Experiment 1: 15<br>Age: 23.33±1.98 yrs.<br>Experiment 2: 12<br>Age: 24.5±2.23 yrs.<br>Experiment 3: 12<br>Age: 22.66±1.82 yrs.	BBS: dynamic balance protocol<br>Power plate(BBS): overall stability index, anteroposterior stability index, mediolateral stability index<br>Isokinetic dynamometer: MVC of all ankle muscle
**Nunes (2016)** [32]	(1) a history of ≥ 2 lateral ankle sprains in the same ankle, with the latest sprain having occurred within 12 months; (2) CAIT (Portuguese) < 23	N: 50<br>Experiment (CAI): 25<br>Age: 22.7±3.3 yrs.<br>Control (Healthy): 25<br>Age: 23.6±3 yrs.	VJT: counter-movement maximum vertical single-leg jump<br>Force plate; time to stabilization, COP displacement (AP/ML) in 3 reference periods (stance/pre-jump/post-jump)
**Oh (2023)** [33]	CAI group<br>(1) ≥ 2 acute LASs required immobilization and/or non-weight bearing for ≥ 3 d's or external supports for ≥ 7 d's or both; (2) history of ≥ 2 'giving way' episodes within the past 6 months; (3) FAAM-ADL < 90%, FAAM-Sports < 80%, and AII ≥ 5<br>Coper group	N: 57<br>Experiment (CAI): 16<br>Age: 22±2 yrs.<br>Experiment (Coper): 16<br>Age: 21±3 yrs.<br>Control (Healthy): 16<br>Age: 22±2 yrs.	SLHS: double-leg anterior jump in the 50% of vertical jump height and landing with single-leg<br>Force plate: anteroposterior stability index, mediolateral stability index, vertical stability index, dynamic postural stability index

Author (Y)	Inclusion criteria	Participants	Tasks and outcome measures
	(1) ≥ 1 acute LASs required immobilization and/or non-weight bearing for ≥ 3 d's or external supports for ≥ 7 d's or both; (2) FAAM-ADL = 100%, FAAM-Sports = 100% and No 'yes' answer on questions 5–9 of the AII		
**Phuaklikhit (2023)** [22]	(1) history of ≥ 1 ankle sprain within 5 years; (2) CAIT < 24	N: 40<br>Age: 20.28±2.45 yrs.	YBT: %normalized reach distance score<br>Accelerometer: at L5 spine level; RMS sway, mean velocity, and jerk of COM
**Rios (2015)** [34]	(1) history of ≥ 2 sprains of the same ankle with ≥ 1 sprain in the last 6 months; (2) sensation of ankle instability presenting as ankle ''giving way'' unilaterally	N: 42<br>Experiment (CAI): 21<br>Age: 25 yrs.<br>Control (Healthy): 21<br>Age: 25 yrs.	SLS: kicking a ball by the other leg on a stable and unstable platform<br>EMG: integral sum of erector spinae, rectus abdominis, Gmed, adductor, biceps femoris, rectus femoris, soleus, medial head of gastrocnemius, lateral head of gastrocnemius, tibialis anterior, peroneus longus<br>Force plate: COP displacement (AP/ML), COP area
**Ross (2004)** [40]#	(1) history of ≥ 1 ankle sprain injury with ≥ 3 days of immobilization with reported ≥ 2 additional ankle sprains; (2) ≥ 2 "giving way" sensations with	N: 28<br>Experiment 1: 14<br>Age: 21.71±2.64 yrs.	VJT: Jump-stabilization maneuver following the completion of single-leg stance testing<br>Force plate: TTS

Author (Y)	Inclusion criteria	Participants	Tasks and outcome measures
	weight-bearing activity within the year prior to their participation.	Control: 14<br>Age: 22±1.92 yrs.	
**Ross (2005)** [41]	(1) history of a severe ankle sprain injury with ≥ 3 days of immobilization with reported ≥ 2 additional ankle sprains; (2) ≥ 2 ‘‘giving way’’ sensations with weight-bearing activity within the year before their participation in this study.	N: 20<br>Experiment 1: 10<br>Age: 22±2.5 yrs.<br>Control: 10<br>Age: 20.8±1.3 yrs.	VJT: 2-footed vertical jump height as they jumped and landed with single-leg<br>Force plate: TTS
**dos Santos (2014)** [35]	(1) history ≥ 2 sprains of the same ankle, including ≥ 1 sprain within the last 6 months; (2) sensation of ankle instability presenting as the ankle ‘‘giving way’’ unilaterally.	N: 42<br>Experiment (CAI): 21<br>Age: 25 yrs.<br>Control (Healthy): 21<br>Age: 25 yrs.	SLS: kicking a ball by the other leg on normal and supinated foot<br>Force plate: COP displacement (AP/ML), 95% confidence intervals for the sway area
**Sierra-Guzmán (2018)** [17]	(1) history of ≥ 1 substantial ankle sprain occurred more than 3 months before study enrollment; (2) ≥ 2 episodes of the ankle ‘‘giving way’’ in the 6 months before the study; (3) CAIT ≤ 24	N: 50<br>Experiment 1: 24<br>Age: 22.4±2.6 yrs.<br>Experiment 2: 24<br>Age: 21.8±12.1 yrs.<br>Control: 24	SEBT (ANT, AM, MED, PM, PL and Composite): mean reach distance<br>BBS: dynamic balance protocol<br>Power plate(BBS): overall stability index, anteroposterior stability index, mediolateral stability index

Author (Y)	Inclusion criteria	Participants	Tasks and outcome measures
		Age: 23.6±3.4 yrs.	
**Shih (2018)** [23]	(1) history of ≥ 1 ankle sprain within the past year with residual symptoms; (2) repetitive ankle sprains within the past year with feelings of 'giving way' within the past 3 months; (3) reporting CAIT score ≤ 27	N: 45<br>Experiment 1 : 15<br>Age: 26.5±4.8 yrs.<br>Experiment 2 : 15<br>Age: 26.9±5.8 yrs.<br>Control : 15<br>Age: 27.9±6.6 yrs.	YBT: %normalized reach distance score<br>EMG: %MVC of peroneus longus, tibialis anterior, and soleus<br>Goniometer: ROM of ankle dorsiflexion
**Simpson (2019)** [36]	(1) history of ≥ 2 lateral ankle sprains with 1 of those lateral ankle sprains occurring more than 3 months and required non-weight bearing activity or immobilization for 24 h; (2) a history of recurrent sprain, the ankle "giving way", or "feelings of instability" on the affected ankle; (3) CAIT ≤ 24	N: 30<br>Experiment (CAI): 15<br>Age: 21.3±1.6 yrs.<br>Control (Healthy): 15<br>Age: 21.5±1.5 yrs.	SLDL: Lateral single-leg drop landing from 25 cm step height<br>Force plate: COP (AP/ML), RMS, velocity and range, time to boundary (AP/ML)
**Simpson (2019)** [37]	(1) history of ≥ 2 lateral ankle sprains with 1 of those lateral ankle sprains occurring more than 3 months and required non-weight bearing activity or immobilization for 24 h; (2) a history of recurrent	N: 30<br>Experiment (CAI): 15<br>Age: 21.3±1.6 yrs.<br>Control (Healthy): 15<br>Age: 21.5±1.5 yrs.	SLDL: anterior step off from 30 cm to the flat at 20-degree inverted platform<br>EMG: %MVIC of tibialis anterior, medial gastrocnemius, peroneus longus, peroneus brevis at initial contact, latency of peroneus longus and

Author (Y)	Inclusion criteria	Participants	Tasks and outcome measures
	sprain, the ankle "giving way", or "feelings of instability" on the affected ankle		brevis, co-contraction index in sagittal plane and frontal plane muscle group<br>3D motion analysis: time to maximum inversion, initial and maximum inversion angle, and maximum inversion velocity
**Steib (2013)** [15]	FAI group<br>(1) ≥ 1 moderate to severe inversion ankle sprain (≥ 8 days sports time loss) within 5 years before study entry; (2) reported ≥2 repeated episodes of spraining or "giving way" within 12 months before testing<br>Coper group<br>(1) reported an initial moderate to severe inversion ankle sprain within 5 years before study entry; (2) no self-reported residual complaints such as pain, swelling, or instability in the involved ankle; (3) ≤ 1 repeated episode of giving way or spraining if all other inclusion criteria were met	N: 57<br>Experiment (FAI): 19<br>Age: 24.95±3.79 yrs.<br>Experiment (Coper): 19<br>Age: 24.53±2.76 yrs.<br>Control (Healthy): 19<br>Age: 23.32±3.79 yrs.	SEBT (ANT/POS/MED/LAT): %normalized reach distance score<br>SLJL: unilateral diagonal jump to the center of force plate<br>Force plate: time to stabilization<br>Isokinetic: joint position sense

Author (Y)	Inclusion criteria	Participants	Tasks and outcome measures
**Tang (2023)** [38]	(1) reported experiencing ankle instability, loss of control, or leg weakness during certain activities; (2) CAIT ≤ 24; (3) suffered ≥ 1 severe ankle sprain with > 1 day of limited mobility, as well as another sprain after the initial injury, with no recent acute sprain within the past 3 months; (4) (-) anterior drawer test and talar tilt test	N: 18<br>Age:<br>M 20.9±1.73 yrs.<br>F 20.3±1.73 yrs.	SLDL: anterior single-leg drop landing from 20 cm above the ground and 5 cm away from the edge of the force plate.<br>Force plate: dynamic postural stability index (anteroposterior stability index, mediolateral stability index, vertical stability index)
**Wikstrom (2010)** [39]	CAI and Coper group<br>(1) history of ≥ 1 unilateral lateral ankle sprain that required immobilization for ≥ 3 days<br>CAI specific criteria<br>(1) ≥ 1 episode of giving way within the past year; (2) ≥ 1 recurrent ankle sprain between 3 and 6 months before study participation; (3) AJFAT > 22<br>Coper specific criteria<br>(1) no pain, weakness, or instability in the involved ankle; (2) AJFAT ≤ 22	N: 72<br>Experiment (CAI): 24<br>Age: 21.7±2.8 yrs.<br>Experiment (Coper): 24<br>Age: 20.8±1.5 yrs.<br>Control (Healthy): 24<br>Age: 21.8±2.6 yrs.	SLHS: double-leg anterior jump in the 50% vertical jump height and landing with single-leg.<br>Force plate: anteroposterior stability index, mediolateral stability index, vertical stability index, dynamic postural stability index
